# Metabolic Network Modularity in Archaea Depends on Growth Conditions

**DOI:** 10.1371/journal.pone.0025874

**Published:** 2011-10-06

**Authors:** Kazuhiro Takemoto, Suritalatu Borjigin

**Affiliations:** 1 PRESTO, Japan Science and Technology Agency, Kawaguchi, Saitama, Japan; 2 Department of Biophysics and Biochemistry, University of Tokyo, Bunkyo-ku, Tokyo, Japan; University of Groningen, Netherlands

## Abstract

Network modularity is an important structural feature in metabolic networks. A previous study suggested that the variability in natural habitat promotes metabolic network modularity in bacteria. However, since many factors influence the structure of the metabolic network, this phenomenon might be limited and there may be other explanations for the change in metabolic network modularity. Therefore, we focus on archaea because they belong to another domain of prokaryotes and show variability in growth conditions (e.g., trophic requirement and optimal growth temperature), but not in habitats because of their specialized growth conditions (e.g., high growth temperature). The relationship between biological features and metabolic network modularity is examined in detail. We first show the absence of a relationship between network modularity and habitat variability in archaea, as archaeal habitats are more limited than bacterial habitats. Although this finding implies the need for further studies regarding the differences in network modularity, it does not contradict previous work. Further investigations reveal alternative explanations. Specifically, growth conditions, trophic requirement, and optimal growth temperature, in particular, affect metabolic network modularity. We have discussed the mechanisms for the growth condition-dependant changes in network modularity. Our findings suggest different explanations for the changes in network modularity and provide new insights into adaptation and evolution in metabolic networks, despite several limitations of data analysis.

## Introduction

Because metabolism is responsible for physiological functions and for maintaining life, it is an important topic not only in general biology but also in applied biological research fields such as biotechnology and medical science. *Metabolism* can be defined as a series of chemical reactions, and it is often represented as a network (called a metabolic network) [1]–[3]. In recent years, several new technologies and high-throughput methods have generated considerable genomic and metabolic network data. Accordingly, comprehensive analyses of metabolic networks have been actively carried out, and the entire picture of metabolic networks has steadily become clearer (reviewed in [4], [5]). Until now, many studies have discussed the mechanisms involved in the evolution of metabolic networks [6]–[8] and environmental adaptation from the viewpoint of metabolism (reviewed in [9], [10]).

When discussing metabolic networks, previous works have focused on *metabolic network modularity* because the network modularity, which reflects how well a network can be decomposed into dense subnetworks that are relatively weakly interconnected, is believed to be one of the important organizing principles of biological networks [11]–[13]. Specifically, Parter et al. [14] revealed that variability in natural habitat promotes metabolic network modularity in bacteria (i.e., the network modularity of an organism living in wider environments is higher), and they showed a mechanism possibly responsible for the change in metabolic network modularity.

However, because previous studies [15]–[19] have reported different structural properties in the metabolic networks between domains and different properties with respect to oxygen requirements and optimal growth temperature, we have 2 natural questions when extending the discussion of network modularity to habitats of species: (i) Are similar results observed in other domains? (ii) Are there other explanations for the differences observed in metabolic network modularity (e.g., can biological features such as oxygen requirements and optimal growth temperature be related to network modularity?)?

**Figure 1:**
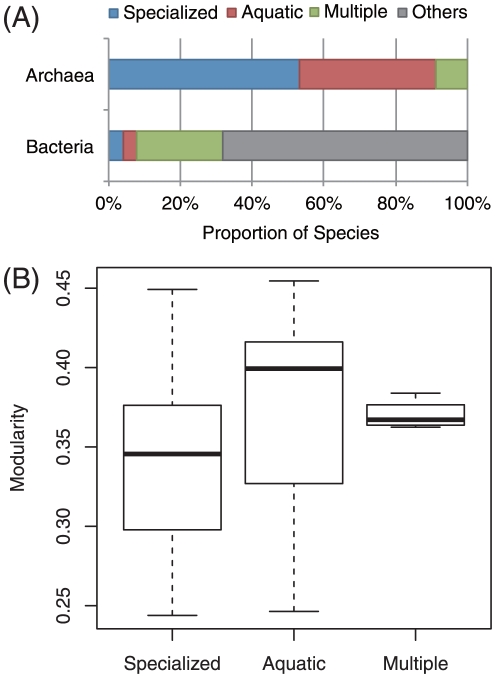
Effect of habitat variability on metabolic network modularity (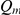). (A) The ratio of species in each category of environmental variability between the archaea and bacteria; values for bacteria were obtained from published data [14]. (B) No relationship was observed between metabolic network modularity and habitat variability in archaea (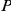-value 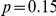, using the Kruskal–Wallis test). The degree of environmental variability increases in the following order: specialized, aquatic, and multiple.

Archaea are interesting examples to consider when answering these questions. Like bacteria, they belong to the prokaryotes and are widely distributed throughout normal and extreme environments (e.g., high temperatures, highly acidic conditions, and oxygen-free conditions) [20], but their habitats are limited (or narrow) due to their specialized growth conditions (see also Figure 1). Thus, it may be possible to discuss other possible mechanisms causing changes in the metabolic network modularity through archaea. Despite this advantage, until now, this evaluation was difficult, because not much genomic and metabolic data were available for archaea because of experimental difficulties. However, the recent genome projects have revealed the whole genomes of many archaea (see [21] for details); moreover, metabolic information has been correctly annotated thanks to the elucidation of the gene manipulation system [22].

In this study, therefore, we investigate other possibilities responsible for changes in the metabolic network modularity in archaea and show 2 main results: The first is that no differences were observed in metabolic network modularity with respect to habitat variability, because of the limited habitats of archaea. The second observation is a change in metabolic network modularity with growth conditions (trophic requirement and optimal growth temperature, in particular) in the absence of habitat variability. This result implies different possible mechanisms of metabolic network modularity, and provides new insights into metabolic network adaptation.

## Results

### Variability in the habitats of archaeal species hardly influences metabolic network modularity

We investigated whether the increase in metabolic network modularity with habitat variability, previously reported in bacteria [14], is observed in archaea.

We selected 45 archaeal species for which biological features and metabolic network data are available (see Table S1 and Materials and Methods for details). Based on previous work [14], we constructed the metabolic networks of archaea whose nodes and undirected edges are metabolites and reactions, respectively, and calculated the metabolic network modularity, 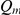 (see Materials and Methods for details). Note that 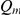 shows no correlation with the number of metabolites (i.e., network size; Spearman's rank correlation coefficient 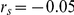 with the 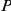-value 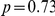) or the number of metabolic links (i.e., reactions; 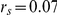, 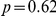) because it was normalized to allow comparison between different network sizes and connectivity. In addition, 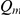 does not correlate with genome size (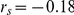 with 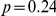) or the number of protein-encoding genes (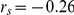, 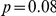), because they are related to the network size and the number of links.

Like a previous study [14], the classification of archaeal lifestyle was determined on the basis of the Entrez Genome Project database [23]. Using this database, 45 archaea were classified into 24 specialized species, 17 aquatic species, and 4 multiple species, where specialized species are organisms living in specialized environments such as marine thermal vents; aquatic species are organisms living in fresh- or seawater environments and are not associated with hosts; and multiple species are organisms living in multiple different kinds of environments, such as species with a wide host range. Note that the lifestyles of organisms are classified into 6 classes in this database (See [14] for the other lifestyle classes) The archaeal lifestyle was weighted in case of specialized and aquatic species, compared that of bacteria (Figure 1A).

Figure 1B shows no statistical difference between habitat variability and metabolic network modularity in archaea. The network modularity of aquatic species seems to be greater than that of specialized species. However, no statistically significant difference is observed because of high variance. The fact that there is no difference in network modularity between aquatic species and specialized species may be not surprising because a previous study also showed a similar result (see also Figure 2 in [14]).

**Figure 2:**
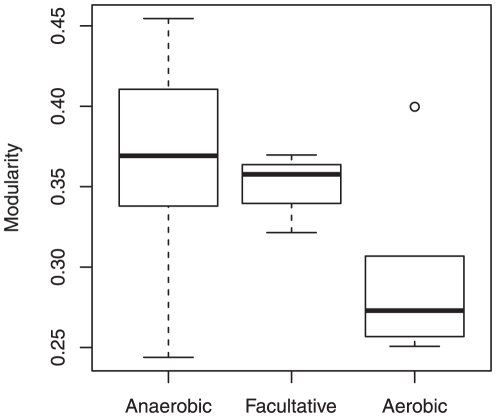
Limited relationship between metabolic network modularity (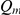) and oxygen requirements. The limited difference in the network modularity with respect to oxygen requirement is observed (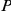-value 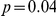, using the Kruskal–Wallis test). The degree of oxygen required increases in the following order: anaerobic, facultative, and aerobic.

However, the fact that multiple species have lower network modularity than aquatic species and specialized species may be remarkable because a greater network modularity is expected as network modularity is promoted by habitat variability (see also Figure 2 in [14]). Note that multiple species have a wider habitat than specialized or aquatic species do. However, the difference between multiple species and the other species is ambiguous because of the small number of multiple species in the sample.

As explained in the previous section, habitat variability hardly explains the differences in metabolic network modularity because of the narrow habitat of archaea species. Are there other explanations for the changes in network modularity? In the following section, we consider other possible explanations for the differences in metabolic network modularity.

### Oxygen requirements have a limited effect on metabolic network modularity

The effect of oxygen on metabolic networks [17], [19] implies a difference in the structure of metabolic network (the network size, in particular) with respect to oxygen requirement. In this section, the effect of oxygen requirements on metabolic network modularity is considered. The 45 archaeal species were classified into 6 aerobes, 3 facultative aerobes, and 36 anaerobes, on the basis of the available literature [24], [25], indicating that the sample is skewed toward anaerobes.

Figure 2 shows that metabolic network modularity seems to slightly decrease with oxygen requirement because there is a small statistically significant difference due to the difference in oxygen requirements (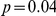, using the Kruskal–Wallis test). Because of the small significance, this result implies a limited effect of oxygen requirements on metabolic network modularity, and it is consistent with the previous works [17], [19], which showed that oxygen requirements hardly affect the topology of metabolic networks excluding the network size.

However, we may be not able to completely reject the effect of oxgen requirement on the network modularity because the difference in network size cannot simply explain the difference in network modularity in addition to the small statistical significance. The network modularity is independent from the network size (see the first subsection in this section). Thus, oxygen requirements may partially contribute the metabolic network modularity although its effect is limited.

### Autotrophs show greater metabolic network modularity than heterotrophs

The reduction in metabolic network modularity due to niche specification [12] suggests that trophic requirement affects network modularity. We investigated the relationship between trophic requirement and network modularity. The 45 archaea were categorized into 22 autotrophs, 7 facultative autotrophs, and 16 heterotrophs, on the basis of the available literature [24], [25].

**Figure 3:**
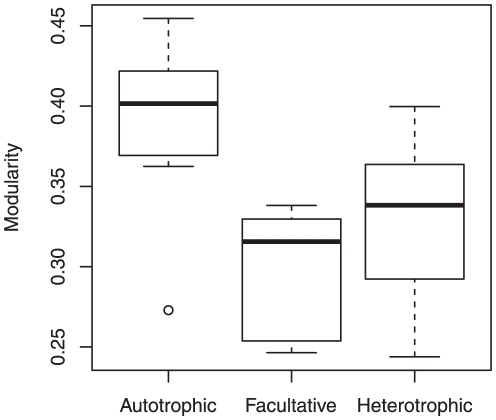
Trophic requirement influences metabolic network modularity (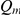). The significant difference in the network modularity with respect to trophic requirement is observed (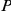-value 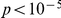, using the Kruskal–Wallis test). The degree of trophic requirement increases in the following order: autotrophic, facultative, and heterotrophic.

We found that the metabolic network modularity of autotrophs is clearly greater than that of facultative autotrophs and heterotrophs (Figure 3). This result suggests another possible explanation for the difference in metabolic network modularity. Most autotrophs are methanogens that generate methane from carbon sources (generally carbon dioxide) under anoxic conditions; thus, it is possible to interpret the difference in network modularity between autotrophs and heterotrophs as the one between methanogens and heterotrophs.

### Metabolic network modularity correlates with optimal growth temperature

Structural differences with respect to optimal growth temperature [18], [19] indicate the effect of temperature on metabolic network modularity. In this section, we investigate the relationship between network modularity and optimal growth temperature. In addition, the effect of optimal growth pH is considered because it is well known that some archaea live in highly acidic environments.

**Figure 4:**
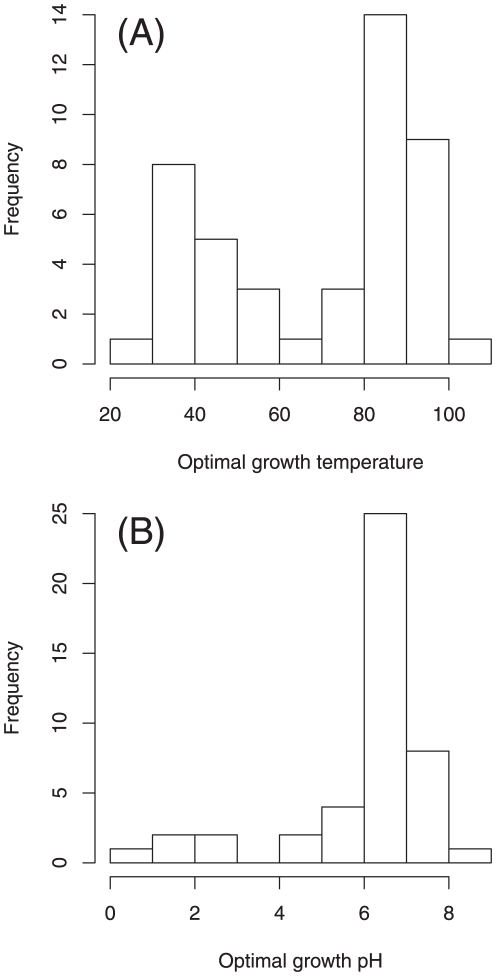
High diversity of archaea based on optimal growth conditions. (A) Optimal growth temperature, (B) Optimal growth pH.

Figure 4 shows the variability in the optimal growth parameters of 45 archaea. The archaea are roughly classified into 2 groups on the basis of optimal growth temperature (Figure 4A): archaea whose optimal growth temperature is around 37

C (generally called mesophiles) and those who optimal growth temperature is around 85

C (generally called hyperthermophiles). The minimum and maximum optimal growth temperatures are 23.4

C and 106

C, respectively. Moreover, most archaea have an optimal growth pH of around 7 (i.e., neutrality); however, some archaea thrive in acidic environments (Figure 4B). The minimum and maximum optimal growth pH values are 0.7 and 9.0, respectively. These results indicate a high diversity of archaea based on optimal growth conditions, but no habitat variability (see Figure 1A).

**Figure 5:**
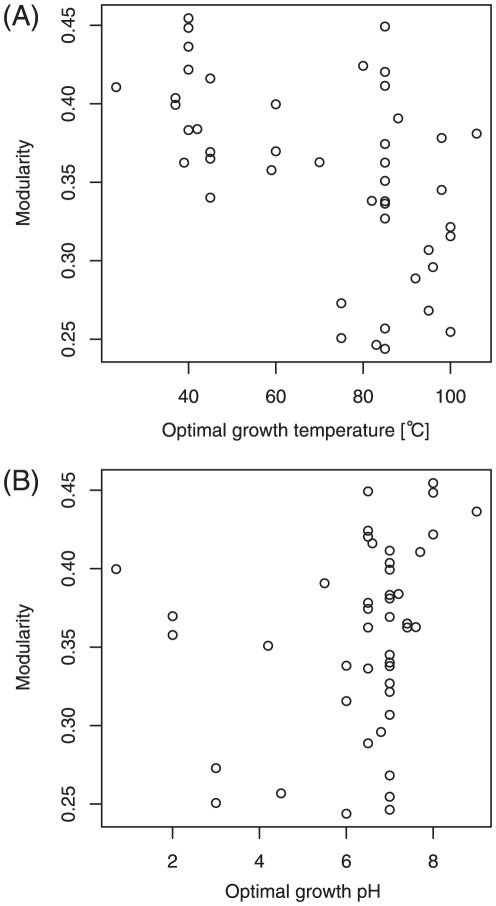
Optimal growth conditions (temperature, in particular) affect metabolic network modularity (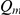). (A) Optimal growth temperature (Spearman's rank correlation coefficient 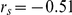 with the 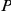-value 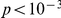), (B) Optimal growth pH (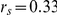 with 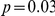)

Figures 5 shows moderate significant correlations between optimal growth conditions and metabolic network modularity. Although there are some outliers, the negative and positive correlations of network modularity are observed in the cases of optimal growth temperature (Figure 5A) and pH (Figure 5B), respectively.

## Discussion

We found no statistically significant differences between habitat variability and metabolic network modularity in archaea (Figure 1B). However, this finding does not contradict the metabolic network modularity promoted by the variability in the natural habitat of an organism in bacteria [14], because the habitat variability of archaea is more limited than that of bacteria. Until now, archaea living in extreme conditions (i.e., species with narrow habitats) have been actively explored because such organisms are useful for industrial applications. Thus, the 45 selected archaea may be weighted toward species with narrow habitats. However, because the existing types of archaea is higher than expected [20], the relationship between network modularity and habitat variability may be observed not only in bacteria but also in archaea when more archaeal metabolic networks are available. Therefore, the effect of growth conditions (i.e., trophic requirement and optimal growth temperature) on network modularity revealed in this study becomes significant in case of organisms with similar habitats (narrow habitats in this study).

Oxygen requirements are likely to reduce metabolic network modularity despite their limited effect (Figure 2). Although the effect of oxygen requirements on network modularity is limited, we discuss how oxygen requirements reduce network modularity. This reduction in network modularity may be explained by using the suggestion by Raymond and Segré [17], i.e., the link rewiring metabolic networks after oxygen becomes available in organisms (i.e., the transition from anaerobe to aerobe). The network modularity indicates that dense subnetworks are weakly connected to one another. If metabolic links are rewired in such networks, the networks may be randomized. As a result, the dense subnetworks may be broken, implying a reduction in network modularity. However, note that the effect of the link rewiring due to the oxygen availability on the network modularity is very limited as shown in Figure 3. The previous studies [17], [19] also show the limited effect of oxygen requirements on structural properties of metabolic networks.

The greater network modularity of autotrophs (Figure 3) may be explained using the implication by Kreimer et al. [12], i.e., the decrease in network modularity due to niche specialization during evolution. Because autotrophs generate essential metabolites (e.g., organic acids and sugars) from very simple carbon sources (generally carbon dioxide), they have metabolic pathways (modules) for carbon fixation. However, the carbon fixation modules become unnecessary when such essential metabolites become enriched in the environment. These metabolic modules may be lost because of its unnecessity during evolution, and organisms might begin to require specific nutrients (i.e., transition to autotrophs to heterotrophs). As a result, metabolic networks might become simplified by the disintegration of dense subnetworks, implying a reduction in metabolic network modularity.

The decrease in metabolic network modularity with respect to optimal growth temperature (and pH) may be discussed using the possible mechanism of the change in network modularity proposed by Parter et al. (see Figure 5 in [14] for details). The authors explained that network modularity decreases as alternative paths between a given metabolite pair vanish in organisms whose habitats are narrow. Such a vanishment of alternative paths indicates that network modules are broken (i.e., a decay of network modularity). However, another work [26] uses the network model to show that such an alternative path vanishes (i.e., is not selected) at a high optimal growth temperature. The selection of alternative paths might be caused by a temperature-dependent selective constraint (negative selection) [27], [28]. Metabolic paths consist of enzymes (i.e., proteins). Because enzymes might need structural stability to survive in hot and/or acidic environments, they tend to easily get deactivated in such conditions, and therefore, the emergence of alternative paths may be restricted. Through this mechanism, metabolic network modularity may decrease with increasing optimal growth temperatures. This mechanism of network modularity change is critically different from those based on species-specific habitats, although these mechanisms state that non-selection of alternative paths between a given metabolite pair may reduce network modularity.

Some outliers are observed in the growth condition-dependent nature of metabolic network modularity. This may be because many biological features, including the focused parameters in this work, intricately influence metabolic network modularity. Thus, it is difficult to determine the most dominant feature for explaining metabolic network modularity at this time, because of the number of samples. Thus, we need to test this growth condition dependence of network modularity using more species although it is difficult at this time because of a few available data on metabolic networks and species phenotypes. Since the development of high-throughput technics may provide more such data, the validation using more species may be possible in the future. In addition to this limitation, our analysis has several other limitations, as do many other works on metabolic network analyses: limited knowledge of metabolic reactions (i.e., missing links), reconstruction of metabolic networks based on genomic information, and failure to consider reaction stoichiometry and direction of reaction (i.e., reversible or irreversible).

Although data analysis has these limitations, the growth condition-dependent nature of network modularity is useful for explaining other possible mechanisms in the change in metabolic network modularity, and they provide new insights into the adaptive and evolutionary mechanisms in metabolic networks.

## Materials and Methods

### Selection of archaeal species

We collected data on oxygen requirements (i.e., aerobic and/or anaerobic), trophic requirement (i.e., autotrophic and/or heterotrophic), optimal growth temperature, and optimal growth pH of archaea based on the available literature [24], [25]. We selected archaea for which data on metabolic networks were available in the KEGG (Kyoto Encyclopedia of Genes and Genomes) database [29], which is a well-known database on metabolic pathways. Moreover, we selected archaea for which data on lifestyles were available in the Entrez Genome Project database [23]. We examined 45 archaeal species.

### Construction of metabolic networks

This part of the research was similar to the previous work [14], and therefore, we could compare the two.

We downloaded XML files (version 0.7.1) containing the metabolic network data of 45 archaea on 20 May 2011 from the KEGG database [29] (ftp://ftp.genome.jp/pub/kegg/xml/kgml/metabolic/organisms/). Note that the KEGG ftp site is available only to paid subscribers beginning July 1, 2011. Based on [14], these metabolic networks are represented by undirected networks (i.e., substrate graphs) in which the nodes and edges correspond to metabolites and reactions (i.e., substrate-product relationships based on atomic mapping [3]), respectively. Ubiquitous metabolites such as H
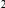
0, ATP, and NADH were removed. Moreover, the largest connect component (or giant component) was extracted from each metabolic network to more accurately calculate network modularity.

### Measurement of metabolic network modularity

This is also similar to the previous work [14], thereby allowing comparison.

To allow the comparison of metabolic network modularity with different network sizes and connectivity, we used the normalized network modularity 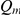 based on [14], defined as
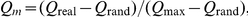



where 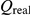 and 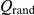 are the network modularity of a real-world metabolic network and the average network modularity value obtained from 300 randomized networks constructed from its real-world metabolic network, respectively. The network modularity measure 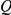 is defined as the fraction of edges that lie within modules rather than between modules relative to that expected by chance (e.g., see Equation (4) in [30] for definition). Each 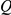 was calculated using the fast greedy algorithm proposed by Clauset et al. [30]. 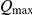 was estimated as 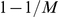, where 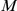 is the number of modules in the real network.

Randomized networks were generated from a real-world metabolic network using the simple edge-rewiring algorithm [31]. This algorithm generates a random network by rewiring 2 randomly-selected edges until the rewiring of all edges is completed. For example, we consider 2 edges: A–B and C–D, where the alphabets and lines are nodes and edges, respectively. Through this edge-rewiring algorithm, in this case, we obtain the edges A–D and C–B (see [31] for details).

In metabolic networks (i.e., substrate graphs), in general, multi-substrate reactions emerge short cycles due to the network representation. For example, we consider a reaction: C1
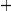
C2
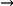
C3
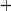
C4. According to the network representation in this work, the cycle of length 4 (the square graph) is generated because the nodes (metabolites) C1 and C2 connect to the nodes C3 and C4. In this manner, cycles are generated when metabolic reactions have multi substrates and multi products. Since these cycles are related to the network modularity, it is not suitable to simply apply this edge-rewiring algorithm to metabolic networks in general. Ideally, randomized networks should be generated with maintenance of the number of short cycles generated due to the network representation. However, this edge-rewiring algorithm does not consider this constraint.

Although the edge-rewiring algorithm has such a limitation, this limitation poses little problem for calculating 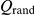 in this work because we used the substrate graphs based on the atomic mapping in which currency metabolites such as water and ATP are neglected. In our metabolic networks, hence, most (about 98% on an average) of metabolic reactions are represented as reactions with single substrate and/or single product as a result. Therefore, short cycles generated due to the network representation hardly arise.

## Supporting Information

Table S1
**A list of 45 archaeal species.** This table includes the species name, KEGG ID (see [32]), genome size, number of protein-encoding genes, lifestyle, oxygen requirements, trophic requirement, optimal growth temperature, and optimal growth pH for each archaeon. In addition, it includes the parameters in each archaeal metabolic network: the number of nodes (i.e., network size), the number of links, 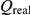, the number of modules (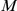), 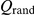, and 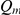.(XLS)
